# Surgical Creation of the Coronary Sinus Orifice in an Octogenarian With Heart Failure

**DOI:** 10.1016/j.atssr.2025.10.019

**Published:** 2025-11-19

**Authors:** Yuichi Yoshida, Shinya Yokoyama, Kozo Kaneda, Shigeo Nagasaka, Hisao Nagato, Noboru Nishiwaki

**Affiliations:** 1 Department of Cardiovascular Surgery, Nagoya City University Graduate School of Medical Sciences, Nagoya, Japan; 2 Department of Cardiovascular Surgery, Takanohara Central Hospital, Nara, Japan; 3 Department of Cardiovascular Surgery, Nagahama City Hospital, Shiga, Japan; 4 Department of Cardiovascular Surgery, Osaka Saiseikai Izuo Hospital, Osaka, Japan

## Abstract

Coronary sinus (CS) ostial atresia is rare, often coexisting with a persistent left superior vena cava (PLSVC), and is exceptional in elderly patients. Right-sided heart failure developed in an 82-year-old man 2 years after aortic valve replacement. Computed tomography and cardiac magnetic resonance imaging showed CS ostial closure and long-segment stenosis at the PLSVC–left brachiocephalic vein junction. At reoperation, a CS neo-ostium was created, followed by ligation of the PLSVC and tricuspid valve replacement. Postoperative imaging showed direct CS drainage into the right atrium, improved coronary venous washout, and reduced left ventricular dimension. Physiologic coronary venous reconstruction may benefit selected octogenarians with CS ostial atresia.

Coronary sinus (CS) ostial atresia is a rare congenital anomaly with few and heterogeneous reports, and evidence in elderly patients remains limited. We describe an octogenarian undergoing CS neo-ostium creation.

An 82-year-old man with a history of aortic regurgitation, tricuspid regurgitation (TR), and atrial fibrillation, with marked left atrial enlargement in the absence of mitral regurgitation, underwent aortic valve replacement with a 25-mm Magna Ease bioprosthesis (Edwards Lifesciences), De Vega tricuspid annuloplasty (calibrated to 27 mm), and left atrial appendage excision 2 years earlier. During the index operation, the CS ostium was found to be imperforate, consistent with membranous atresia. Postoperative echocardiography demonstrated only modest reverse remodeling of the left ventricle, with the left ventricular end-diastolic dimension (LVDd) decreasing from 63 to 57 mm. He was managed medically with low doses of a loop diuretic and a mineralocorticoid receptor antagonist.

Two years later, he presented with worsening right-sided heart failure, including scrotal hydrocele and lower limb cellulitis. He was admitted with stable vital signs (body surface area, 1.79 m^2^): blood pressure, 118/64 mm Hg; heart rate, 76 beats/min; and oxygen saturation, 97%. Laboratory test results showed hemoglobin level of 12.8 g/dL, platelet count of 191 × 10^9^/L, creatinine concentration of 1.07 mg/dL, and B-type natriuretic peptide level of 166 pg/mL.

Transthoracic echocardiography (TTE) revealed severe TR due to annular dilation and leaflet tethering, with large pleural effusions and ascites on ultrasound. LVDd was 60 mm with a preserved ejection fraction (64%) and normal wall motion, without mitral regurgitation or paravalvular leak. Cardiac magnetic resonance imaging confirmed membranous closure of the CS ostium and no CS–right atrium communication (Figure 1A). Computed tomography (CT) demonstrated marked right atrial enlargement (85 × 85 mm) with a right ventricular basal diameter of 58 mm; the left atrium remained massively dilated (100 × 120 mm), unchanged from imaging 2 years earlier. Three-dimensional CT showed a markedly dilated great cardiac vein with minimal coronary venous opacification (Figures 1C, 1D) and a long-segment stenosis at the persistent left superior vena cava (PLSVC)–left brachiocephalic vein (LBCV) junction (Figures 1B, 1C).

**Figure 1:**
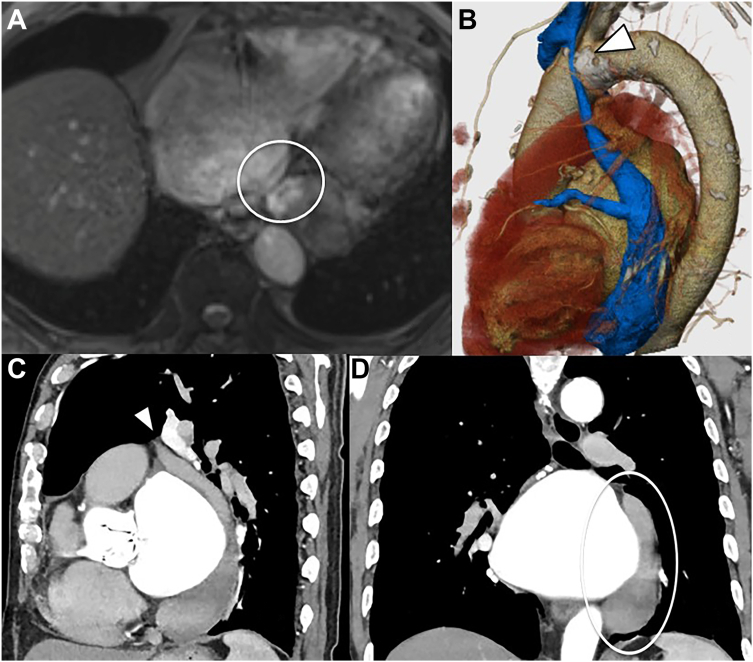
(A) Cardiac magnetic resonance image shows membranous closure of the coronary sinus ostium with no direct coronary sinus–right atrium communication (circle). Computed tomography (B) 3-dimensional, (C) sagittal, and (D) coronal views show long-segment stenosis at the persistent left superior vena cava–left brachiocephalic vein junction (B, C; arrowheads), with a markedly dilated great cardiac vein (D; oval) and near-absence of venous opacification (C, D).

Coronary angiography showed no significant coronary artery stenosis and was inconclusive regarding coronary venous drainage. Preoperative right-sided heart catheterization showed pulmonary capillary wedge pressure of 10/10 (mean, 9) mm Hg, pulmonary artery pressure of 26/12 (mean, 17) mm Hg, right ventricular pressure of 30/3 mm Hg (end-diastolic, 8 mm Hg), and right atrial pressure of 7/8 (mean, 6) mm Hg; no oximetry step-up was observed. Cardiac vein pressure could not be obtained because stenosis at the PLSVC–LBCV junction precluded catheter passage.

Despite escalation of guideline-directed therapy, peripheral edema improved; however, echocardiography revealed persistent severe TR, prompting reoperation. Because CS ostial atresia could further compromise coronary venous drainage, it was addressed concomitantly.

Reoperation was performed through median sternotomy. Cardiopulmonary bypass was established with bicaval venous drainage and ascending aortic cannulation. After aortic cross-clamping, cardioplegic arrest was achieved with antegrade blood cardioplegia.

Vertical right atriotomy exposed thebesian veins in the right atrium. A septal incision allowed inspection of the mitral valve, where additional tiny ostia were noted near the posterior annulus. Test cardioplegia produced subtle seepage. Accordingly, the right atrial endocardium was incised at the commissure between the septal and posterior tricuspid leaflets, just inferior to the fossa ovalis and away from the apex of the triangle of Koch, to create a CS neo-ostium (Figure 2). Careful dissection within the interatrial septum exposed fatty tissue, beneath which a cordlike fibrous structure was identified. Trial puncture of this structure yielded dark venous blood, confirming entry into the coronary venous system. A 15-mm neo-ostium was then created and sutured to the right atrial endocardium with 6 interrupted 6-0 polypropylene sutures. Epicardially, the cardiac vein was firm; even on incision, its wall remained rigid, unlike systemic veins. The PLSVC was then ligated at its junction with the LBCV, and cardioplegia return confirmed adequate coronary venous drainage.

**Figure 2:**
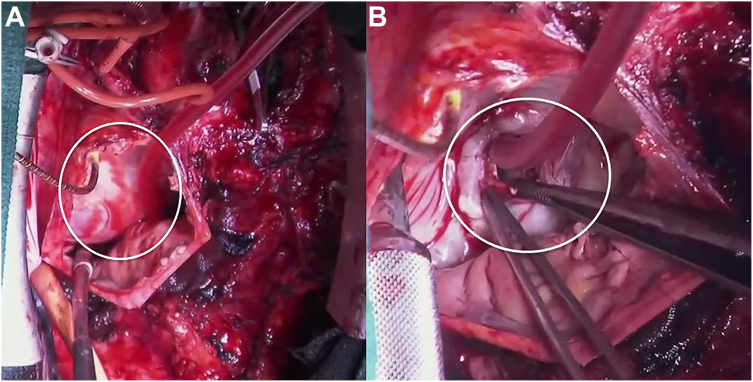
(A) View demonstrating membranous atresia at the expected site of the coronary sinus ostium (circle). (B) After recanalization, a 15-mm neo-ostium was created and sutured to the right atrial endocardium (circle).

After creation of the CS neo-ostium, tricuspid valve replacement was performed under cardioplegic arrest for precise suturing near conduction tissue. The prior De Vega annuloplasty sutures were partially disrupted, and a 29-mm Mosaic bioprosthesis (Medtronic) was implanted. The septotomy and atriotomy were then closed. Cardiopulmonary bypass and cross-clamp times were 233 minutes and 117 minutes, respectively. Intrinsic rhythm returned after unclamping; given proximity to the conduction system, a prophylactic permanent right ventricular pacing lead was placed.

TTE postoperatively showed a decrease in LVDd from 60 to 52 mm, with preserved ejection fraction of 59%. Cardiac magnetic resonance imaging confirmed direct CS drainage into the right atrium (Figure 3A). Multiplanar reformatted CT clearly demonstrated improved coronary venous washout of the great cardiac vein (Figure 3B). The patient remained asymptomatic during 18-month follow-up.

**Figure 3:**
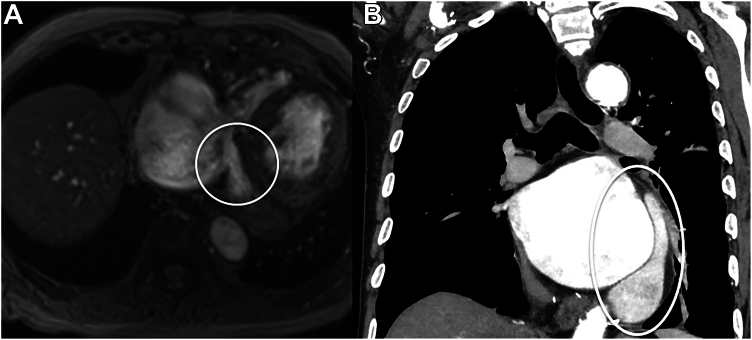
(A) Magnetic resonance imaging shows reopening of the coronary sinus ostium with direct right atrial drainage (circle). (B) Coronal computed tomography shows improved coronary venous washout (oval).

## Comment

CS ostial atresia with venous drainage through the PLSVC is a rare congenital anomaly characterized by absence of the normal CS ostium, and most patients remain asymptomatic.^1^ Although traditionally considered mainly in single-ventricle physiology and Fontan circulation, CS ostial atresia in elderly patients is exceedingly uncommon, and evidence is scarce. In our case, a long-segment PLSVC-LBCV stenosis likely impeded coronary venous drainage, prompting targeted restoration. Experimental work and clinical observations suggest that coronary venous obstruction can contribute to myocardial congestion.^2^^,^^3^ CS ostial atresia was recognized at aortic valve replacement but left untreated because aortic regurgitation predominated; subsequent heart failure highlighted its clinical impact and the need not to overlook CS ostial atresia at the index operation. Even at reoperation, identifying the CS was challenging, and light-guided endoscopic localization was precluded by the long-segment PLSVC-LBCV stenosis.^4^

Postoperative imaging provided unique functional evidence of restored coronary venous drainage, with CT showing improved washout in the great cardiac vein. Physiologically, the volume of coronary venous drainage to the right atrium would not be expected to change after CS ostium creation, and left ventricular preload should even increase once severe TR is corrected. Nevertheless, postoperative TTE showed a reduction in LVDd with preserved ejection fraction, supporting relief of coronary venous congestion.

This case highlights a broader principle of physiologic reconstruction: restoring venous drainage by re-creating or redirecting pathways, analogous to creation of a neo-ostium in complex atrioventricular valve repair and anatomic redirection in unroofed CS or partial anomalous pulmonary venous return repair. Surgical creation of a CS ostium can be successfully performed even in an octogenarian, with potential implications for biventricular function.
